# Admission immune-inflammatory biomarker profiles for trauma severity stratification: an externally validated machine learning study

**DOI:** 10.3389/fimmu.2026.1950721

**Published:** 2026-10-14

**Authors:** Xianling Dai, Ligai Zhang, Zhiyu Wu, Hanqing Xu, Bin Chen, Yang Zhou, Ye Xiao, Yu Wu, Shiqing Xiang, Jian He, Jing Bao, Yuting Huang

**Affiliations:** 1 Department of Clinical Laboratory Medicine, First Affiliated Hospital, Army Medical University, Chongqing, China; 2 Department of Clinical Laboratory Medicine, Chongqing Banan Traditional Chinese Medicine Hospital, Chongqing, China; 3 Health Management Center, First Affiliated Hospital, Army Medical University, Chongqing, China; 4 Department of Respiratory and Critical Care Medicine, First Affiliated Hospital, Army Medical University, Chongqing, China

**Keywords:** external validation, immune-inflammatory biomarkers, injury severity, machine learning, trauma

## Abstract

**Background:**

Traumatic injury induces rapid systemic immune-inflammatory alterations, but whether routinely measurable admission biomarkers can provide an early biological estimate of anatomical injury burden remains uncertain. This study aimed to characterize trauma-associated immune-inflammatory profiles across trauma severity levels defined by injury severity score (ISS) and evaluate their combined value for early severity stratification.

**Methods:**

This two-center retrospective study included healthy participants as a physiological reference group, non-trauma acute-care patients as a clinically relevant comparator, and trauma patients classified as mild (ISS <16), moderate (ISS 16–25), or severe (ISS >25). Nineteen admission laboratory biomarkers and demographic variables were evaluated in a development cohort of 775 participants and an independent external validation cohort of 349 participants. Multinomial least absolute shrinkage and selection operator regression was used to identify severity-associated biomarkers. Ten classifiers were compared on the same predefined trauma-only development split, and the selected model was evaluated in an independent external trauma cohort.

**Results:**

A 10-feature panel was retained following feature selection and collectively defined an immune-inflammatory profile associated with increasing trauma severity. Increasing injury burden was associated with heterogeneous and partly nonlinear inflammatory and hematologic alterations, including changes in neutrophil-related indices, lymphocyte-related measures, and albumin. The multinomial logistic regression model achieved the best overall performance, with an accuracy of 0.941 and 0.973 in the internal holdout and external validation cohorts, respectively, with an external macro-AUC of 0.993. Model interpretation consistently identified neutrophil count and RDW-CV as major contributors to severity stratification.

**Conclusion:**

Admission-stage blood biomarkers capture reproducible immune-inflammatory alterations associated with increasing anatomical injury severity. Their combined analysis may provide a complementary approach for early biological assessment of injury burden and supports further investigation of clinically accessible biomarkers of trauma-associated immune-inflammatory dysregulation.

## 1 Introduction

Trauma remains a major cause of death and disability worldwide, particularly among young and working-age populations (1–4). Rapid assessment of injury severity is essential for prioritizing diagnostic resources, guiding resuscitation, and identifying patients who require intensive monitoring or urgent intervention. Anatomical scoring systems, particularly the Injury Severity Score (ISS), are widely used to quantify overall injury burden and provide important prognostic information (5, 6). However, definitive anatomical characterization frequently depends on imaging, operative findings, and comprehensive injury documentation. During the early phase of evaluation, additional biological indicators may therefore provide complementary information regarding the systemic consequences of traumatic injury.

Traumatic tissue damage initiates an immediate and complex immune-inflammatory response. Damage-associated molecular patterns released from injured cells activate innate immune pathways and stimulate the production of inflammatory mediators (7). These early biological alterations may contain valuable information regarding injury burden and subsequent clinical deterioration, creating an opportunity for biomarker-based severity stratification before imaging findings become available. Accumulating evidence suggests that trauma severity is accompanied by coordinated immune-inflammatory dysregulation involving activation of innate immune responses, suppression of adaptive immunity, and systemic metabolic disturbances. Several inflammatory biomarkers, including C-reactive protein (CRP), interleukin-6 (IL-6), and procalcitonin (PCT), have been reported to increase proportionally with injury severity and correlate with adverse clinical outcomes (8–10). Routine blood tests, including complete blood counts, coagulation profiles, and serum chemistry panels, provide additional objective measures of immune and metabolic status (11). Ratios derived from immune cell populations, such as neutrophil-to-lymphocyte ratio (NLR), platelet-to-lymphocyte ratio (PLR), and lymphocyte-to-monocyte ratio (LMR), have demonstrated predictive value for complications and mortality in trauma cohorts (12, 13). Similarly, markers of nutritional and hematologic status, including albumin and red cell distribution width (RDW), have been linked to poor outcomes and prolonged recovery following injury (14). However, most previous studies have evaluated individual biomarkers or specific clinical outcomes, whereas trauma-related immune-inflammatory alterations are multidimensional and potentially nonlinear. It therefore remains unclear whether routinely available admission biomarkers can collectively define a reproducible biological profile associated with increasing anatomical injury severity.

Multivariable predictive modeling provides a practical framework for integrating demographic characteristics, inflammatory mediators, hematologic parameters, and immune-related indices that may collectively reflect trauma severity. Both conventional statistical models and machine-learning approaches have increasingly been applied to trauma-related risk stratification and outcome prediction, with their relative advantages depending on data structure, sample size, and clinical context (15). A systematic review of machine-learning models for trauma outcomes identified substantial heterogeneity in model development and highlighted the limited use of external validation and formal calibration, underscoring that high model complexity alone does not ensure clinical generalizability (16). In traumatic brain injury, machine-learning approaches such as support vector machines, random forests, and neural networks showed promising performance for mortality and unfavorable-outcome prediction (17). More broadly, multidimensional models incorporating clinical, immunological, and cytokine data have been used to predict post-traumatic complications, while recent externally validated trauma models further emphasize the importance of transportability across institutions (18). Together, these findings support direct comparison of statistical and machine-learning approaches under identical analytical conditions, with model selection guided not only by discrimination but also by robustness, interpretability, calibration, and external validation.

In this study, we investigated admission-stage immune-inflammatory profiles in three distinct roles: healthy participants served only as a physiological reference, non-trauma patients provided a more clinically relevant comparator, and trauma patients formed the primary mild–moderate–severe classification task. Predictor-selection stability was evaluated across these analytical contexts, and 10 statistical and machine-learning classifiers were compared using an identical patient split and predictor panel. The best-performing model was subsequently assessed in an independent external-validation cohort. We hypothesized that admission biomarkers would provide a complementary early biological estimate of anatomical injury burden before definitive ISS assignment.

## 2 Methods

### 2.1 Data collection

We conducted a retrospective cohort study of 1,678 participants from two centers (**Figure 1A**). The development cohort was assembled at the First Affiliated Hospital of Army Medical University and the external cohort at Chongqing Banan Traditional Chinese Medicine Hospital. Exclusion criteria included HIV/AIDS, malignancies, pre-trauma use of steroids (any dosage or administration route) or immunomodulatory medications, incomplete laboratory parameters. Laboratory values were excluded only when they were non-numeric, explicitly flagged as analytically invalid in the source record, or incompatible with the recorded measurement unit. Clinically extreme but technically valid values were retained. Monthly distributions of patients stratified by ISS levels are illustrated in **Supplementary Figure S1**. The final development cohort comprised 775 participants (184 healthy, 91 non-trauma and 500 trauma), whereas the external cohort comprised 349 participants (61 healthy, 28 non-trauma and 260 trauma). Healthy participants were retained only for physiological-reference analyses, and non-trauma patients were retained as a clinically relevant comparator. Neither group entered the primary trauma-only classifier.

**Figure 1:**
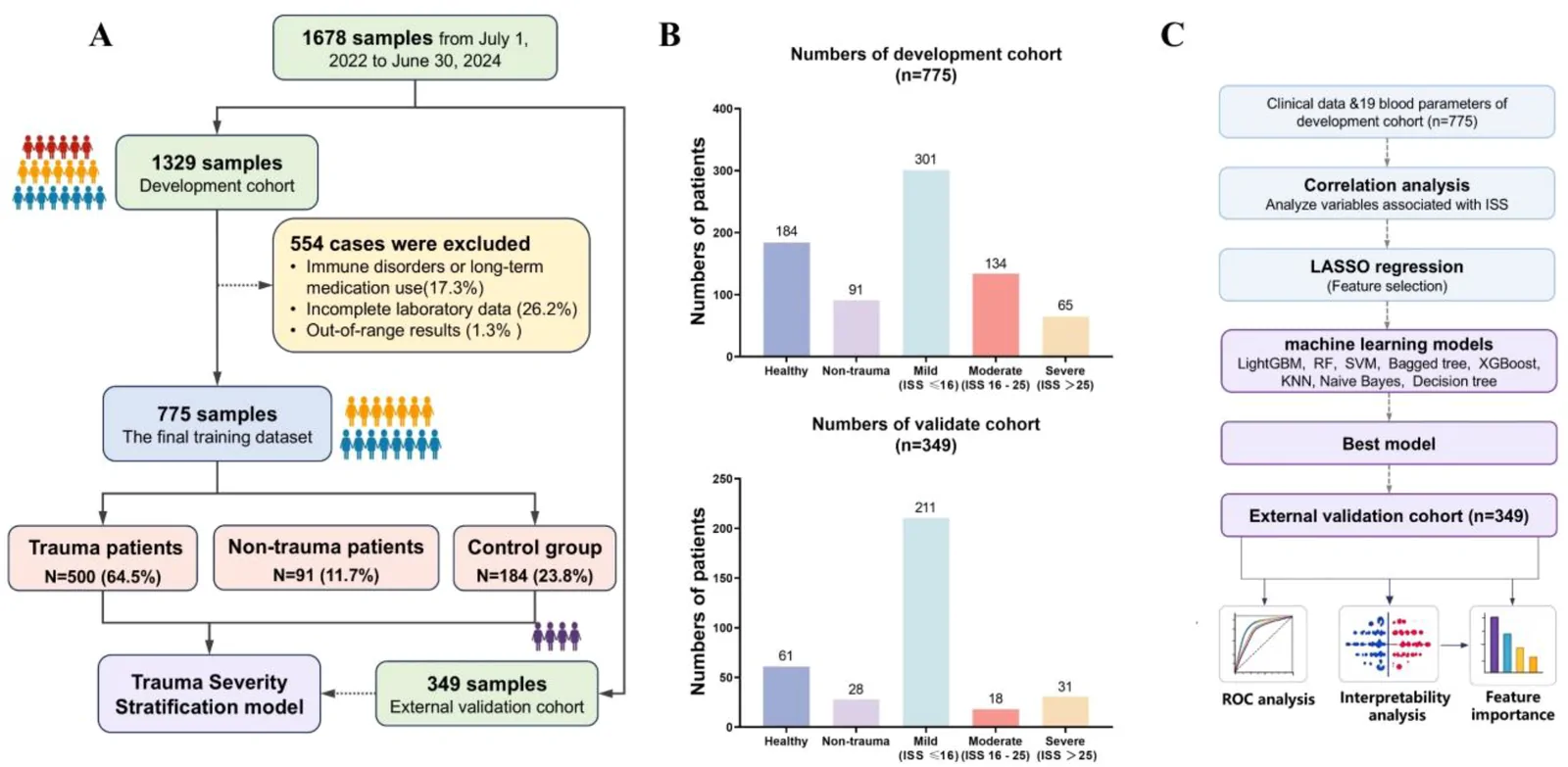
Study design and cohort overview **(A)** Participant flow and exclusions; **(B)** Distribution of ISS groups; **(C)** Study design, cohort composition, and analytical workflow.

For the primary modeling analysis, 500 development trauma patients with the requisite ISS information were retained in the previously fixed split (399 training and 101 internal holdout). External validation used 260 trauma patients. The internal holdout subset was used for model selection, including comparisons of candidate algorithms, predictor panels, and class-balancing strategies. It is therefore described as an internal model-selection and evaluation cohort rather than an independent test set. The final model specification was fixed before evaluation in the independent external cohort of 260 trauma patients. This study was approved by the Ethics Committee of the First Affiliated Hospital, Army Medical University (KY2023140), which waived informed consent for retrospective anonymized data.

### 2.2 Routine blood parameters

The earliest available laboratory result obtained within 24 h after hospital admission was retained. When multiple results were available, only the first was used to reduce the influence of treatment, resuscitation, and transfusion. The 19 candidate laboratory biomarkers were albumin (Alb), white blood cell count (WBC), platelet count, neutrophil count, lymphocyte count, monocyte count, platelet-to-lymphocyte ratio (PLR), neutrophil-to-lymphocyte ratio (NLR), lymphocyte-to-monocyte ratio (LMR), red cell distribution width-coefficient of variation (RDW-CV), and red cell distribution width-standard deviation (RDW-SD). Additionally, five arterial blood gas parameters were assessed: pH, partial pressure of carbon dioxide (pCO2), partial pressure of oxygen (pO2), lactate, and oxygen saturation (SaO2). Three systemic inflammatory biomarkers, C-reactive protein (CRP), procalcitonin (PCT), and interleukin-6 (IL-6), were included.

### 2.3 Injury characteristics

Trauma severity was classified using the ISS, with injury data retrospectively extracted from discharge reports. According to the abbreviated injury scale (AIS) guidelines, injuries were categorized into six anatomical regions: head/neck, face, thorax, abdomen, extremities, and external structures. The ISS was calculated as the sum of the squares of the highest AIS scores in the three most severely injured body regions; if any injury had an AIS score of 6, the ISS was automatically assigned a value of 75 (19). For stratification, ISS scores <16 were classified as mild trauma (Level I), scores of 16–25 as moderate trauma (Level II), and scores >25 as severe trauma (Level III), consistent with established clinical thresholds (6, 20).

### 2.4 Model construction

Ten statistical and machine-learning classification models were developed to predict ISS levels using biochemical parameters, with the objective of achieving optimal predictive performance. Models included multinomial logistic regression (multinomial LR), ordinal logistic regression (ordinal LR), bagged trees, extreme gradient boosting (XGBoost), light gradient boosting (LightGBM), decision trees, a Naive Bayes classifier, k-nearest neighbors (KNN), random forest (RF), and a support vector machine (SVM). Accuracy, balanced accuracy, macro-F1, multiclass Matthews correlation coefficient (MCC), macro-AUC, sensitivity, specificity and confusion matrices were reported. Implementation was performed in R version 4.6.1(R Foundation for Statistical Computing, Vienna, Austria). Model-specific implementations and specifications are provided in **Supplementary Table S4**. Class-stratified 10-fold cross-validation was performed exclusively within the training set for multinomial LASSO penalty selection. All preprocessing and feature-selection steps were derived from the training data, while the fixed internal holdout set and external-validation cohort remained untouched. No logarithmic or other nonlinear transformation was applied to skewed predictors before modeling. Where standardization was required, centering and scaling parameters were estimated from the training data only and subsequently applied to the internal holdout and external-validation datasets.

Because the training cohort contained unequal numbers of mild, moderate, and severe trauma cases, we examined whether class imbalance materially affected the performance of the selected model. Synthetic minority over-sampling technique (SMOTE) was used as a controlled resampling technique to generate training datasets with progressively more balanced class distributions. SMOTE was implemented using custom R code following the standard nearest-neighbor interpolation procedure. The number of nearest neighbors was set to 5, and all resampling procedures were reproducible under the prespecified random-seed scheme initiated with seed = 2026. The original training distribution comprised 240 mild, 109 moderate, and 50 severe cases. Three balancing conditions were evaluated: moderate SMOTE (240/160/120), strong SMOTE (240/200/180), and full SMOTE (240/240/240) for mild, moderate, and severe trauma, respectively. Continuous predictors were standardized using means and standard deviations estimated exclusively from the original training data. Synthetic observations were subsequently generated in the standardized training feature space using 5 within-class nearest neighbors. Synthetic observations were not recursively reused as neighbors. The internal hold-out and external-validation cohorts retained their natural class distributions and were not resampled. Models fitted under the original and three progressively balanced training distributions were evaluated in the same 101-patient internal hold-out cohort. The principal criteria were balanced accuracy, macro-F1, MCC, and moderate- and severe-class recall.

As an exploratory analysis addressing discrimination beyond healthy controls, we additionally evaluated trauma vs non-trauma classification using the final multinomial LR framework (equivalent to binary logistic regression for this two-class outcome). A routine eight-variable panel (age, albumin, platelet count, neutrophil count, monocyte count, PLR, RDW-CV, and RDW-SD) was compared with panels adding CRP, PCT, or IL-6 individually and with an inflammation-enhanced panel containing all three markers. Decision-curve analysis treated trauma as the event and calculated net benefit across threshold probabilities of 0.05-0.95 relative to treat-all and treat-none strategies.

## 3 Results

### 3.1 Patient characteristics and laboratory parameters

A total of 1,678 participants were screened, yielding 775 participants in the development cohort and 349 in the external cohort (**Figure 1A**). The development cohort comprised 184 healthy participants, 91 non-trauma patients, and 500 trauma patients. Trauma patients were stratified according to ISS into mild injury (ISS<16, n=301), moderate injury (ISS 16–25, n=134), and severe trauma (ISS>25, n=65). The external cohort comprised 349 participants, of whom 260 trauma patients constituted the independent external-validation sample for the primary trauma-severity model. Baseline characteristics of the development and validation cohort are summarized in **Supplementary Table S1** and the distributions of ISS categories in both cohorts are summarized in **Figure 1B**. Mild trauma represented the largest subgroup in both development and validation cohorts, whereas severe trauma accounted for a comparatively smaller proportion of cases, reflecting the inherent class imbalance commonly observed in real-world emergency trauma datasets. To establish an admission-stage trauma severity stratification framework, demographic variables and 19 routine blood biomarkers were integrated into a machine learning workflow (**Figure 1C**). The analytical pipeline included correlation analysis, least absolute shrinkage and selection operator (LASSO) based feature selection, and comparative evaluation of ten statistical and machine-learning algorithms, followed by external cohort validation and model interpretability analysis.

The global distributions of laboratory biomarkers across healthy participants, non-trauma patients and ISS groups are illustrated in **Figure 2C** and **Supplementary Figures S2**-**S4**. Several inflammatory and immune-related biomarkers differed substantially across trauma severity strata. WBC, neutrophil count, NLR, monocyte count, platelet count, and PLR peaked in the moderate group and declined in the severe group, whereas IL-6, PCT, CRP, RDW-CV, RDW-SD, and lactate showed clearer increases toward severe trauma. Albumin and lymphocyte counts were lower in severe trauma. These findings indicate heterogeneous and partly nonlinear biomarker patterns across ISS categories rather than a uniformly progressive inflammatory response. PCO2 and pO2 showed elevated levels in trauma patients compared with non-trauma controls, although these parameters demonstrated less pronounced monotonic progression across ISS subgroups. Clinical laboratory parameters for the patient cohorts are detailed in **Supplementary Table S2**, **S3**. Notably, several biomarkers, including neutrophil, monocyte and RDW-CV showed marked separation among trauma subgroups, supporting their potential utility for early trauma severity discrimination.

**Figure 2:**
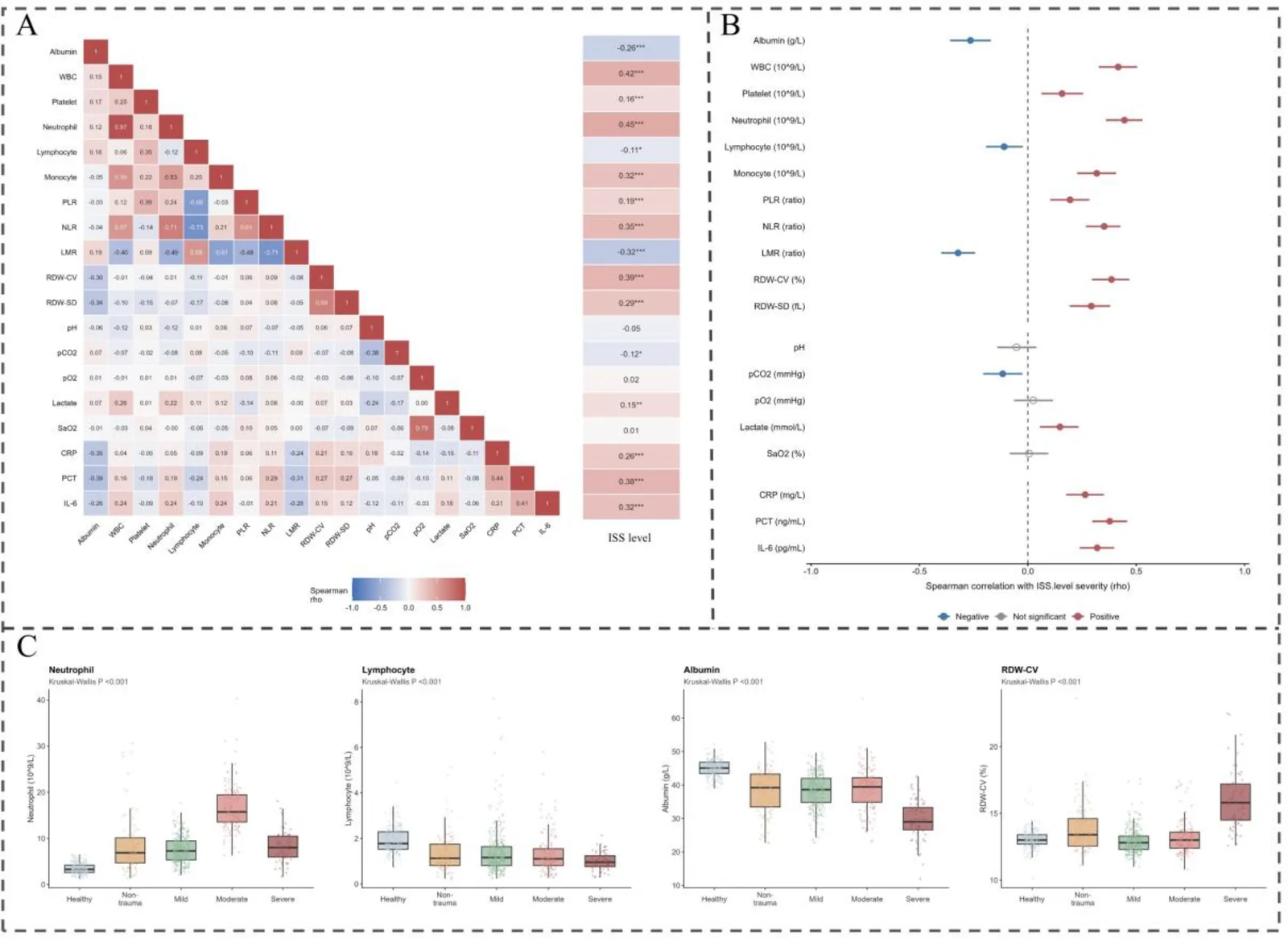
Admission biomarker distributions and correlations with trauma severity. **(A)** Pairwise Spearman correlation matrix for the candidate biomarkers and ISS level; **(B)** Spearman correlations between each admission biomarker and ISS level, with point estimates and 95% confidence intervals; **(C)** Representative distributions of neutrophil count, lymphocyte count, albumin, and RDW-CV across healthy, non-trauma acute-care, mild, moderate, and severe groups.

Correlation analyses further demonstrate strong associations between multiple laboratory biomarkers and ISS level (**Figures 2A, B**). Using ISS level as an ordered severity index, the largest positive Spearman correlations were observed for neutrophil count (r=0.45), WBC (r=0.42), RDW-CV (r=0.39), PCT (r=0.38), NLR (r=0.35), monocyte count (r=0.32), and IL-6 (r=0.32). Albumin (r=−0.26), LMR (r=−0.32), lymphocyte count (r=−0.11), and pCO2 (r=−0.12) were inversely associated with severity. Platelet count (r=0.16), PLR (r=0.19), RDW-SD (r=0.29), lactate (r=0.15), and CRP (r=0.26) showed positive associations, whereas pH, pO2, and SaO2 were not clearly associated. These findings indicate heterogeneous systemic inflammatory and hematologic profiles associated with injury severity, including higher neutrophil-related indices in some severity strata and reduced peripheral lymphocyte counts in more severely injured patients.

### 3.2 Construction of trauma severity model and external validation

To identify predictors that were robustly retained across different analytical contexts, we evaluated three controlled training contexts while holding the trauma sample, trauma outcome, candidate biomarkers, and LASSO settings constant (21, 22). Context A combined the 399 trauma patients with 147 healthy participants (n=546; Healthy, Mild, Moderate, and Severe) and assessed selection against a physiological reference. Context B combined the same trauma patients with the 72 non-trauma training subset (n=471; Non-trauma, Mild, Moderate, and Severe) and provided a clinically relevant comparator. Context C contained only 399 trauma training patients (Mild, Moderate, and Severe) and assessed selection for severity discrimination. All three analyses used the same 16 candidate predictors, including 14 blood-based variables and two demographic characteristics (age and sex). Five blood-gas parameters were excluded for the weak correlations with trauma severity in the preceding analysis.

In Context B, coefficient trajectories progressively converged toward zero with increasing penalization parameter λ, indicating gradual elimination of less informative variables during feature regularization (**Supplementary Figure S5**). Multinomial LASSO retained all 16 predictors at λ_min_=0.00781 and 13 predictors at λ_1se_=0.02386 (**Figure 3A**). Because a single cross-validation solution can be unstable when predictors are correlated, we repeated selection in 500 class-stratified bootstrap resamples within each context. Ten predictors—age, albumin, platelet count, neutrophil count, monocyte count, PLR, RDW-CV, RDW-SD, CRP, and PCT—were selected at λ_1se_ with more than 80% of resamples in every context (**Figures 3B, C**). By contrast, LMR, lymphocyte count, IL-6, sex, and NLR were context dependent, whereas WBC did not reach the stability threshold in any context. In particular, several immune-related predictors were selected less consistently when healthy participants were replaced by non-trauma patients, indicating that the apparent importance was sensitive to comparator composition. The ten cross-context stable predictors were therefore carried forward as the parsimonious primary variables, while the complete 16-feature set was retained as a sensitivity comparator. Notably, several selected biomarkers, including neutrophil-related indices and inflammatory mediators, were consistent with the correlation analyses shown in **Figure 2**, reinforcing their biological relevance for injury severity stratification.

**Figure 3:**
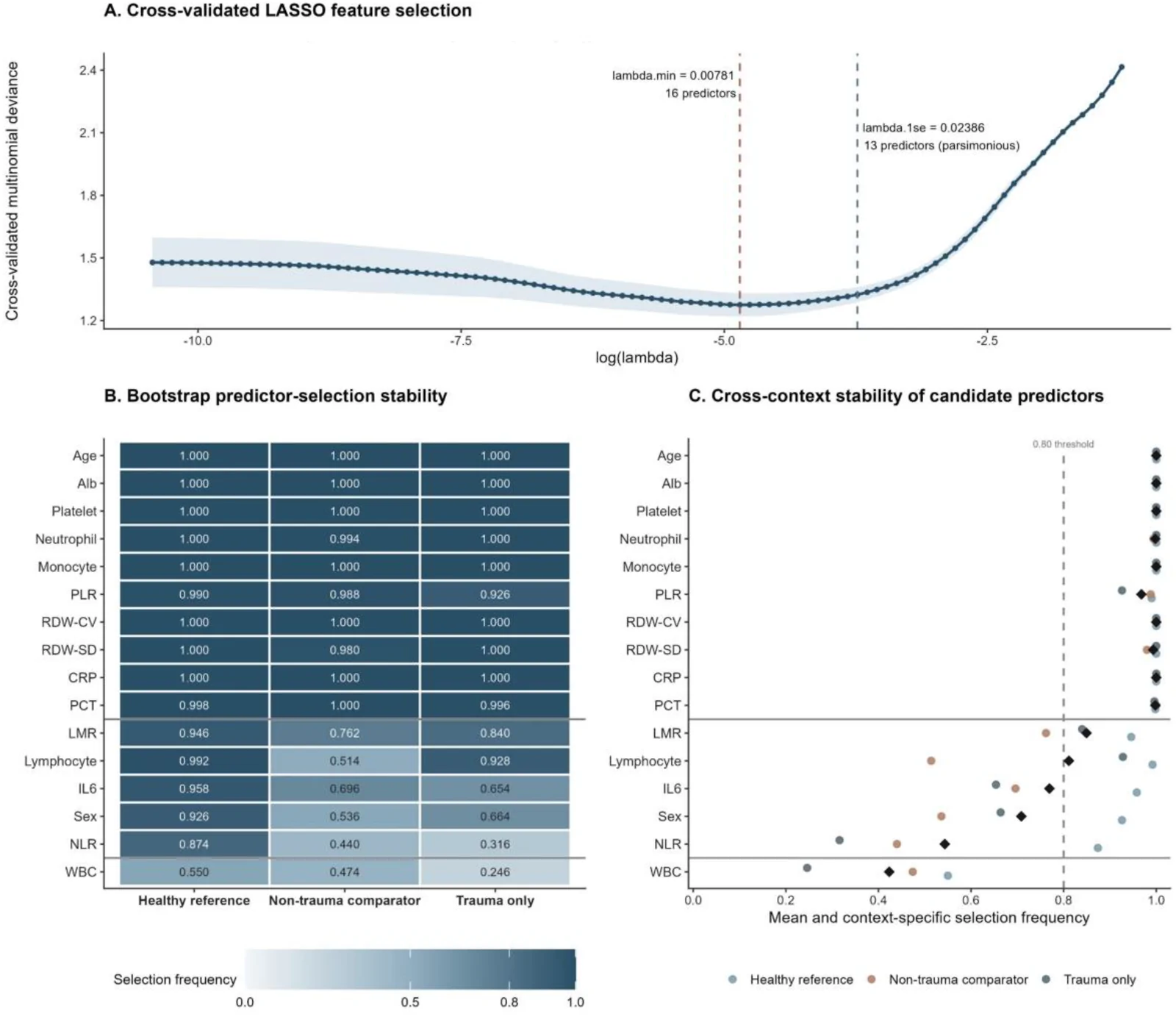
Feature selection and stability across analytical contexts. **(A)** Cross-validated multinomial deviance of LASSO in function of the penalty parameter λ in the non-trauma comparator context; **(B)** Selection frequencies at λ_1se_ from 500 class-stratified bootstrap resamples in healthy reference, non-trauma comparator, and trauma-only training contexts; **(C)** Mean cross-context selection frequency (diamonds) together with individual context-specific frequencies; the dashed line denotes the prespecified 0.80 stability threshold.

We first compared the ten candidate algorithms in the trauma-only training context using the original development data, including multinomial LR, ordinal LR, LightGBM, RF, SVM, Bagged Tree, XGBoost, KNN, Naive Bayes, and Decision Tree classifiers. Class-stratified 10-fold cross-validation was performed within the training set for multinomial LASSO penalty selection. Candidate classifiers were subsequently fitted using the prespecified model specifications summarized in **Supplementary Table S4**. Internal-holdout and external-validation observations were not used for model specification or fitting. Model performance metrics are summarized in **Figure 4A**. Multinomial LR showed the best observed class-balanced performance, with an accuracy of 0.941, balanced accuracy of 0.951, macro-F1 of 0.940, MCC of 0.894, and macro-AUC of 0.990. SVM ranked next (balanced accuracy 0.919, macro-F1 0.907, MCC 0.841), whereas LightGBM achieved 0.879, 0.889, and 0.823, respectively. Ordinal LR performed least well (balanced accuracy 0.598, macro-F1 0.623, MCC 0.452). Although trauma severity is intrinsically ordered, ordinal LR imposes a relatively restrictive structure in which each predictor is assumed to exert a broadly consistent effect across successive severity thresholds. In the present data, however, admission biomarkers may contribute differently to distinguishing mild from moderate trauma than to distinguishing moderate from severe trauma. Multinomial LR does not impose this common-effect constraint and instead allows class-specific predictor coefficients, providing greater flexibility to capture heterogeneous biomarker patterns across severity categories. This greater flexibility may explain its markedly better performance than ordinal LR in our cohort. Multinomial LR was therefore retained as the candidate modeling framework for the subsequent development-stage comparisons.

**Figure 4:**
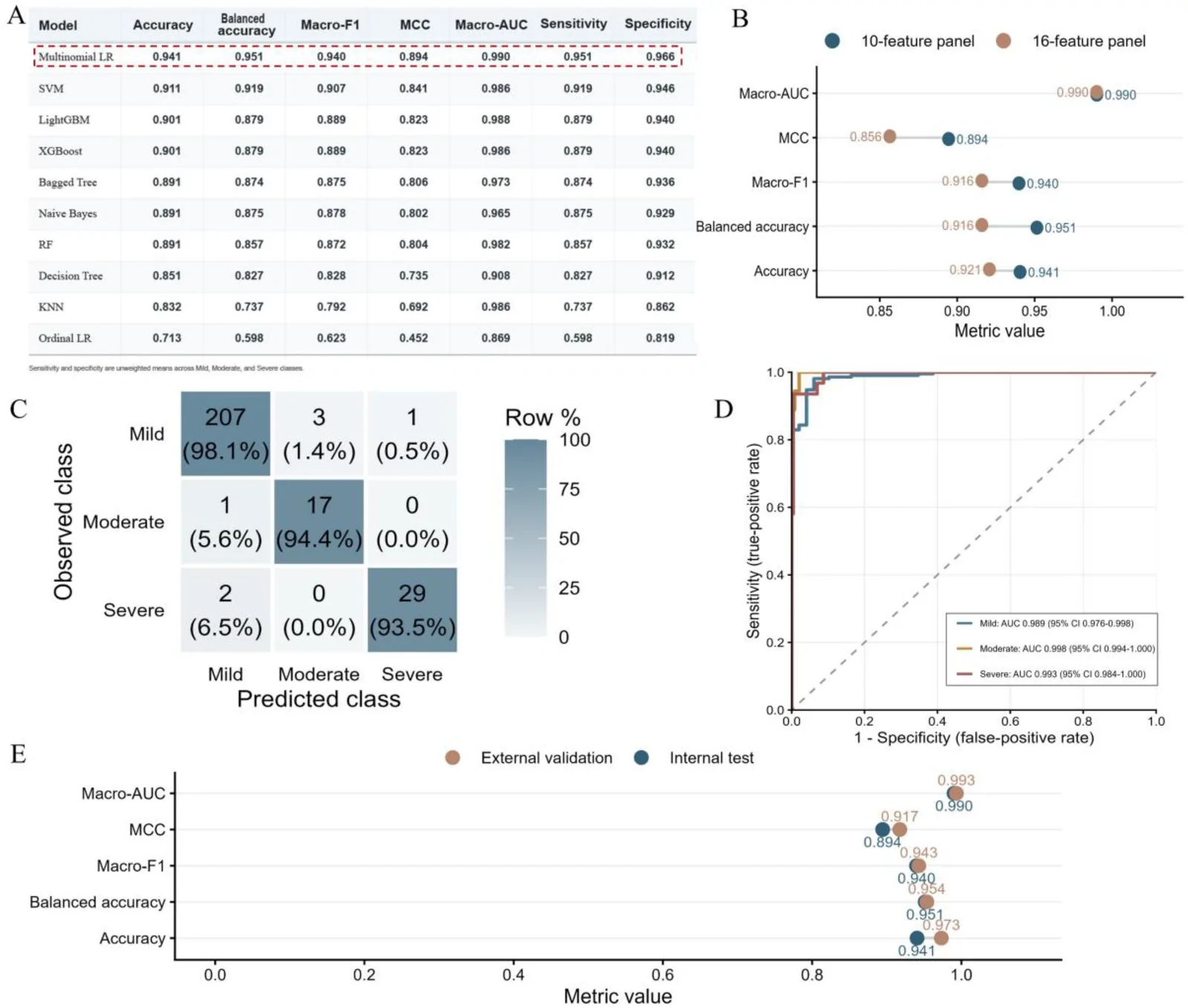
Development and external validation of the trauma severity model. **(A)** Internal-holdout comparison of ten algorithms using identical variables; **(B)** Internal comparison of 10- and 16-feature multinomial LR models; **(C)** External-validation confusion matrix for 260 trauma patients; **(D)** External one-vs-rest ROC curves for mild, moderate, and severe trauma; **(E)** Internal and external overall metrics. Sensitivity and specificity in panel **(A)** are macro-averaged across classes.

With the selected multinomial LR framework, we next compared the stability-selected 10-feature variables against the complete 16-feature variables using multinomial LR and the identical patient split (**Figure 4B**). The 10-feature model achieved higher balanced accuracy (0.951 versus 0.916), macro-F1 (0.940 versus 0.916), and MCC (0.894 versus 0.856), while macro-AUC was 0.990 for both panels. For the 10-feature model, internal-holdout recall was 0.934 for mild, 0.920 for moderate, and 1.000 for severe trauma, with 57/61, 23/25, and 15/15 patients correctly classified, respectively (**Supplementary Figure S6**). These findings supported retention of the more parsimonious 10-feature variables.

We next examined whether the unequal training-class distribution affected the performance of the selected 10-predictor multinomial logistic regression model. Starting from 240 mild, 109 moderate, and 50 severe training cases, SMOTE was used to generate moderate, strong, and full balanced training distributions. Progressive correction of the class imbalance did not alter the predicted class of the 101 internal holdout patients. As shown in **Supplementary Figure S8A**, balanced accuracy (0.951), macro-F1 (0.940), MCC (0.894), and class-specific recalls were unchanged. Macro-AUC ranged from 0.990 to 0.991, and the multiclass Brier score ranged from 0.106 without SMOTE to 0.101 with full SMOTE (**Supplementary Figure S8C**). Because synthetic oversampling did not improve class discrimination and provided only a small numerical change in probability performance (**Supplementary Figure S8B**), the model fitted without SMOTE was retained as the more parsimonious primary specification. Thus, the final development-stage model was a 10-feature multinomial LR model trained on the original class distribution.

After the model type, predictor panel, and class-balancing strategy had been fixed, the final model was evaluated in the external cohort. The trained multinomial LR model achieved an accuracy of 0.973, balanced accuracy of 0.954, macro-F1 of 0.943, MCC of 0.917, and macro-AUC of 0.993 (95% CI 0.986–0.999) in 260 external trauma patients (**Figures 4C–E**). Recall was 0.981 for mild (207/211), 0.944 for moderate (17/18), and 0.935 for severe trauma (29/31). One-vs-rest AUCs were 0.989 (95% CI 0.976–0.998), 0.998 (0.994–1.000), and 0.993 (0.984–1.000), respectively. The unrecalibrated multiclass Brier score was 0.106 (95% CI, 0.037-0.194) internally and 0.055 (95% CI, 0.023-0.092) externally. One-versus-rest calibration intercepts and slopes varied across severity classes, and several calibration slopes were below 1, indicating that high discrimination did not translate into fully calibrated class probabilities. Class-specific precision, sensitivity, specificity, F1 scores, and one-versus-rest AUCs, together with confusion matrices, are reported in **Supplementary Tables S5**, **S6** and **Supplementary Figures S6**, **S7**.

Moreover, to assess whether the final classifier depended on inflammatory biomarkers that may not be rapidly available, we compared the retained 10-feature multinomial model with nested reduced panels using the identical predefined development split and modeling procedure (**Supplementary Table S8**). IL-6 was not discussed because it was excluded in the retained model. In the internal holdout cohort, this no-PCT model achieved a balanced accuracy of 0.924, macro-F1 of 0.917, MCC of 0.859, macro-AUC of 0.989, and severe-trauma sensitivity of 0.933, compared with 0.951, 0.940, 0.894, 0.990, and 1.000, respectively, for the full CRP-plus-PCT model. In the external cohort, the corresponding no-PCT model retained balanced accuracy of 0.929, macro-F1 of 0.918, and severe-trauma sensitivity of 0.871, whereas the full model achieved 0.954, 0.943, and 0.935, respectively. Removing both CRP and PCT produced larger reductions, particularly externally, where balanced accuracy decreased to 0.841 and severe-trauma sensitivity to 0.677. These results show that a reduced model retained substantial discriminative performance when PCT was unavailable, while CRP and PCT together retained complementary information for multiclass discrimination and severe trauma recognition.

In the exploratory trauma vs non-trauma task, the routine eight-variable logistic model achieved balanced accuracy 0.605, macro-F1 0.639, MCC 0.428, AUC 0.681, and non-trauma recall 0.211. Adding IL-6 produced the largest improvement in class performance (balanced accuracy 0.632, macro-F1 0.676, MCC 0.481, and non-trauma recall 0.263). Adding all three inflammatory markers yielded balanced accuracy 0.627, macro-F1 0.665, MCC 0.424, AUC 0.722, and non-trauma recall 0.263. The routine and inflammation-enhanced models provided greater net benefit than treat-all and treat-none over 54 and 73 of the 91 evaluated thresholds, respectively (**Supplementary Figure S9**; **Supplementary Table S7**).

### 3.3 Interpretation of the trauma severity model

With mild trauma as the reference class, the standardized multinomial coefficients identified different conditional signatures for moderate and severe injury (**Figure 5A**). For moderate versus mild trauma, neutrophil count (beta=14.65, FDR-adjusted P = 0.003), monocyte count (beta=8.01, FDR-adjusted P = 0.003), and PLR (beta=5.41, FDR-adjusted P = 0.005) showed the largest positive coefficients. For severe versus mild trauma, the largest positive coefficients were observed for RDW-CV (beta=11.01, FDR-adjusted P = 0.005), RDW-SD (beta=9.06, FDR-adjusted P = 0.018), and CRP (beta=6.86, FDR-adjusted P = 0.003). Albumin showed a negative severe-versus-mild coefficient (beta=-4.29), although this association did not remain below the 0.05 threshold after multiplicity adjustment (FDR-adjusted P = 0.076).

**Figure 5:**
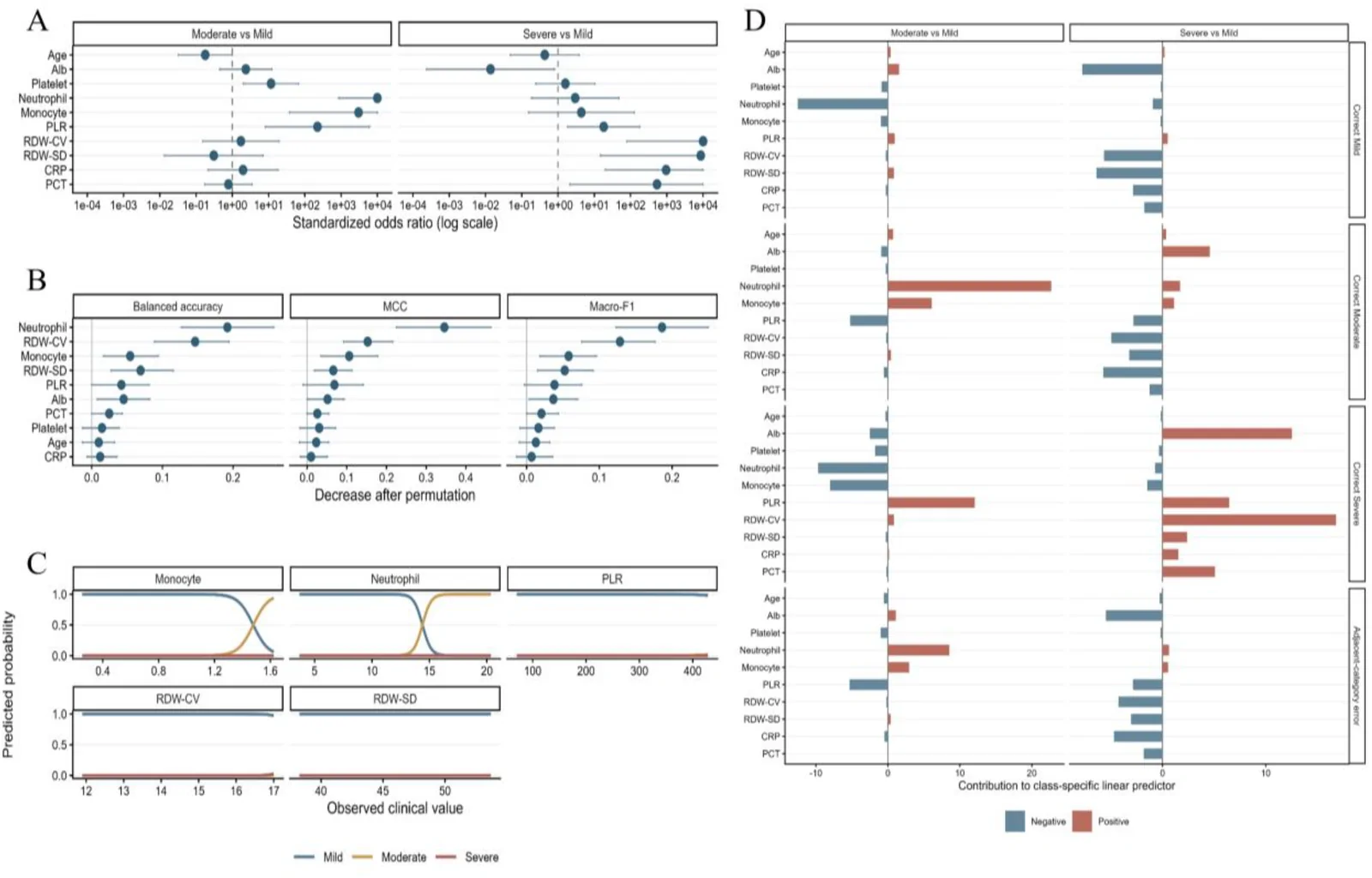
Interpretation of the final 10-feature multinomial LR model **(A)** Standardized class-specific multinomial regression coefficients for moderate versus mild and severe versus mild trauma; **(B)** Internal-holdout permutation importance from 500 repetitions; **(C)** Predicted-probability profiles across selected predictor ranges while other predictors were fixed at training medians; **(D)** Additive contributions for representative correctly classified cases and an adjacent-category error.

Permutation analysis quantified how much each predictor contributed to discrimination in the unchanged 101-patient internal holdout set (**Figure 5B**). Across 500 repetitions, permuting neutrophil count produced the largest mean reduction in macro-F1 (0.186; 95% permutation interval 0.123-0.249), followed by RDW-CV (0.128; 0.076-0.176), monocyte count (0.058; 0.019-0.096), and RDW-SD (0.052; 0.015-0.092). The mean macro-F1 reductions were smaller for PLR (0.038), albumin (0.037), PCT (0.020), platelet count (0.016), age (0.013), and CRP (0.007), and the intervals for several of these smaller contributions included zero. The ordering was reasonably preserved in the external cohort. Neutrophil count and RDW-CV remained the two highest-ranked predictors, and the internal and external macro-F1 importance rankings had a Spearman correlation of 0.794. CRP contributed more strongly externally than internally, illustrating that conditional importance can vary with cohort composition even when overall discrimination is maintained.

The probability profiles further separated the roles of the dominant predictors (**Figure 5C**). Increasing neutrophil and monocyte counts and PLR shifted probability primarily from mild toward moderate trauma when the remaining predictors were fixed at their training medians, whereas increasing RDW-CV and RDW-SD shifted probability toward severe trauma. These profiles were evaluated only over the 5th-95th percentile training ranges and therefore do not define clinical cut points. In the patient-level decompositions (**Figure 5D**), the representative correctly classified mild, moderate, and severe cases each received a predicted probability exceeding 0.999 for the observed class. By contrast, the illustrated adjacent-category error was an observed moderate case assigned to mild trauma, with probabilities of 0.912 for mild and 0.088 for moderate; its combined predictor contributions were therefore positioned on the mild side of the decision boundary. Together, the coefficient, permutation, response-profile, and patient-level analyses show how the model combined distinct inflammatory and erythrocyte-distribution signals.

## 4 Discussion

In this two-center retrospective study, we characterized admission-stage immune-inflammatory alterations across increasing trauma severity and evaluated their combined stratification value using an interpretable framework. A stability-selected 10-feature multinomial LR model provided strong discrimination in both internal and external trauma cohorts. In the exploratory binary analysis using the same regression framework, IL-6 produced the largest single-marker improvement in internal classification performance, and the three immune-inflammatory marker panel showed internal decision-curve net benefit across most evaluated thresholds. The findings indicate that early laboratory profiles contain complementary biological information that may be available while definitive imaging, operative findings, and injury documentation are still being assembled.

A notable finding of this study was the dominant contribution of neutrophil-associated biomarkers to trauma severity stratification. Across correlation analyses, LASSO feature selection, and interpretability analyses, neutrophil count consistently emerged as the most influential predictor. Trauma-induced neutrophilia reflects rapid activation of the innate immune system following tissue injury and has been closely associated with systemic inflammatory response, endothelial dysfunction, secondary infection risk, and organ injury progression (23). The strong predictive contribution of neutrophil count observed in the current study therefore aligns well with the known pathophysiological mechanisms underlying post-traumatic immune dysregulation.

In contrast, CRP exhibited relatively weaker predictive contribution despite showing significant elevation across ISS groups, likely because CRP elevation typically occurs several hours after trauma onset and may therefore be less sensitive during the immediate admission phase (24). RDW-related parameters, particularly RDW-CV and RDW-SD, demonstrated important contributions to model performance. Elevated RDW has previously been associated with hemorrhage severity, hypoxia, oxidative stress, and systemic inflammation, suggesting that erythrocyte heterogeneity may represent an additional surrogate marker of trauma-associated physiological disturbance.

Across controlled comparisons, multinomial LR outperformed the more complex tree-based models and the ordinal LR model on the internal holdout set. This finding indicates that greater model complexity (25–29) did not translate into improved discrimination in the present cohort and supports the use of multinomial LR as a more parsimonious and interpretable framework. Relative to the 16-feature candidate set, the 10-feature model achieved higher balanced accuracy, macro-F1, and MCC than the 16-feature model in the internal holdout set. Class-specific results did not indicate collapse toward the majority mild class. Moderate and severe recalls were 0.920 and 1.000 internally and 0.944 and 0.935 externally. Most errors involved adjacent mild and moderate categories, consistent with continuous biological variation across fixed ISS cut points. However, the model should not be considered a replacement for imaging, anatomical assessment, or established trauma scoring systems. Rather, it may provide a complementary biological estimate of anatomical injury burden before definitive ISS assignment. Early identification of patients may have potential value for prioritizing further diagnostic evaluation and informing the allocation of emergency and critical-care resources.

Several limitations remain. First, patients with complete laboratory measurements were selected for the primary analysis to ensure that model development was based on observed rather than extensively reconstructed data. In this retrospective cohort, missingness was unlikely to be purely random, because the ordering of biomarkers such as IL-6, PCT, and arterial blood-gas tests may have depended on clinicians’ assessment of injury severity and clinical need. Moreover, incomplete cases often lacked several variables simultaneously, making multiple imputation highly dependent on model assumptions and potentially introducing additional uncertainty into downstream feature selection and predictive modeling. This complete-case approach therefore provided a more conservative analytical framework, although it may also have introduced selection bias and limited the representativeness of the final cohort (30). Second, exact injury-to-blood-collection times were inconsistent, although the earliest result within 24 h of admission was used, and time-dependent kinetics may differ between centers. Third, only two centers were included and the moderate external subgroup was small. Differences in laboratory platforms and analytical workflows, together with incompletely recoverable historical analyzer and reference-range metadata, may have contributed to between-center dataset shift and limit model transportability. The class-imbalance analysis indicated that the performance of the multinomial model was not materially dependent on synthetic balancing of the development training data. Nevertheless, the limited number of severe cases means that the apparent robustness to class imbalance should be confirmed in larger cohorts. Fourth, although the predefined ISS categories provide clinically interpretable severity strata, converting a continuous score into fixed classes inevitably reduces information and may exaggerate differences between patients located near adjacent cutoffs (31). The poorer performance of ordinal LR in the present study suggests that simply imposing an ordered-class structure does not fully resolve this limitation. Future work using continuous ISS modeling may better preserve the underlying anatomical severity gradient. Fifth, despite strong discrimination, several class-specific calibration slopes were substantially below 1, indicating over-extreme predicted probabilities. The model should therefore be regarded primarily as a discrimination tool at this stage, and prospective validation with assessment of recalibration is required before clinical deployment. The incremental value of the model beyond information already available to the treating trauma team at the time of blood sampling was not evaluated. Its potential use before definitive anatomical assessment therefore remains hypothetical and requires prospective validation, including assessment of whether laboratory results are available early enough to inform clinical decision-making.

## 5 Conclusions

In conclusion, admission-stage immune-inflammatory and hematologic profiles were associated with increasing anatomical trauma severity. Cross-context stability analysis identified a parsimonious 10-feature predictor panel, and multinomial LR provided strong class-balanced discrimination in both internal and external cohorts. Neutrophil count and RDW-related measures contributed prominently to model discrimination, although these associations should not be interpreted causally. The proposed framework may provide complementary biological information during early trauma assessment, but prospective multicenter studies are required to determine its incremental clinical utility beyond established anatomical evaluation.

## Data Availability

The raw data supporting the conclusions of this article will be made available by the authors, without undue reservation.
