# Supplementary material for: Admission immune-inflammatory biomarker profiles for trauma severity stratification: an externally validated machine learning study
Source: Front Immunol. 2026 Oct 14;17:1950721. doi: 10.3389/fimmu.2026.1950721 (PMC13713292; doi:10.3389/fimmu.2026.1950721)
Supplement: Supplementary file 1 [file SupplementaryFile1.docx]

**Admission Immune-Inflammatory Biomarker Profiles for Trauma Severity Stratification: An Externally Validated Machine Learning Study**

Xianling Dai^1 §^, Ligai Zhang^1 §^, Zhiyu Wu^3 §^, Hanqing Xu^1^, Bin Chen^1^, Yang Zhou^1^, Ye Xiao^2^, Yu Wu^1^, Shiqing Xiang^1^, Jian He^4*^, Jing Bao^1*^ and Yuting Huang^1*^

^1^ Department of Clinical Laboratory Medicine, First Affiliated Hospital, Army Medical University, Chongqing 400038, China.

E-mail:[yutinghuang@tmmu.edu.cn](mailto:yutinghuang@tmmu.edu.cn)

^2^ Health Management Center, First Affiliated Hospital, Army Medical University, Chongqing 400038, China.

^3^ Department of Clinical Laboratory Medicine, Chongqing Banan Traditional Chinese Medicine Hospital 400055, China.

^4^ Department of Respiratory and Critical Care Medicine, First Affiliated Hospital, Army Medical University, Chongqing 400038, China.

^§^X.D., L.Z. and Z.W. contributed equally to the manuscript.

*Y. H., J.B. and J.H. were the corresponding authors

**Figure S1** Bar chart of the number of different ISS levels trauma patients involved monthly at Southwest Hospital from 1 July 2022 to 30 June 2024.

**Table S1** Basic demographics of patients

| Southwest Hospital (n=775) | | | | | | |
| --- | --- | --- | --- | --- | --- | --- |
| Trauma (n=500) | | | | | Non-trauma (n=91) | Health (n=184) |
|  | Mild | Moderate | Severe | | Non-trauma | Health |
| Counts | n=301 | n=134 | n=65 | | n=91 | n=184 |
| Male | n=213 (70.8%) | n=102 (76.1%) | n=42 (64.6%) | | n=67 (73.6%) | n=113 (61.4%) |
| Female | n=88 (29.2%) | n=32 (23.9%) | n=23 (35.4%) | | n=24 (26.4%) | n=71 (38.6%) |
| Mean age [range] | 49 [13-75] | 40 [10-69] | 52 [13-70] | | 60 [14-72] | 44 [21-69] |
| Injury region | | | | | | |
| Head/neck injury | | | | n=260 (52.0%) | | |
| Face injury | | | | n=99 (19.8%) | | |
| Chest injury | | | | n=320 (64.0%) | | |
| Abdomen or pelvic contents injury | | | | n=172 (34.4%) | | |
| Extremities or pelvic girdle injury | | | | n=260 (52.0%) | | |
| External injury | | | | n=150 (30.0%) | | |
| Chongqing Banan Traditional Chinese Medicine Hospital (n=349) | | | | | | |
| Trauma (n=260) | | | | | Non-trauma (n=28) | Health (n=61) |
|  | Mild | Moderate | Severe | | Non-trauma | Health |
| Counts | n=211 | n=18 | n=31 | | n=28 | n=61 |
| Male | n=97 (46.0%) | n=15 (83.3%) | n=19 (61.3%) | | n=16 (57.1%) | n=49 (80.3%) |
| Female | n=114 (54.0%) | n=3 (16.7%) | n=12 (38.7%) | | n=12 (42.9%) | n=12 (19.7%) |
| Mean age [range] | 68 [12-97] | 59 [15-89] | 74 [32-94] | | 59 [18-96] | 43 [17-68] |
| Injury region | | | | | | |
| Head/neck injury | | | | n=102 (39.2%) | | |
| Face injury | | | | n=53 (20.4%) | | |
| Chest injury | | | | n=127 (48.8%) | | |
| Abdomen or pelvic contents injury | | | | n=86 (33.1%) | | |
| Extremities or pelvic girdle injury | | | | n=186 (71.5%) | | |
| External injury | | | | n=54 (20.8%) | | |

**Table S2** Laboratory distributions in the development cohort

| Variable | Unit | Injury level | | | Non-trauma (n=91) | Health  (n=184) |
| --- | --- | --- | --- | --- | --- | --- |
|  |  | Mild (n=301) | Moderate (n=134) | Severe (n=65) |  |  |
| IL-6 | pg/mL | 21.02  (11.28-56.02) | 47.22  (23.50-95.49) | 51.60  (26.06-160.50) | 40.50  (13.05-91.85) | 4.33  (3.61-5.06) |
| PCT | ng/mL | 0.08  (0.05-0.33) | 0.26  (0.07-0.54) | 0.60  (0.35-3.25) | 0.13  (0.06-0.70) | 0.02  (0.02-0.04) |
| CRP | mg/L | 20.10  (7.47-50.90) | 25.75  (7.92-65.25) | 96.90  (36.30-165.00) | 41.65  (10.13-101.17) | 0.87  (0.41-1.72) |
| LMR | ratio | 2.8±3.03 | 1.27±0.89 | 1.95±1.26 | 2.38 ± 1.97 | 6.17±2.01 |
| PLR | ratio | 181.3±104.57 | 269.89±179.44 | 216.37±151.45 | 220.70 ± 167.62 | 115.83±33.19 |
| NLR | ratio | 8.04±6.69 | 17.59±12.25 | 9.74±5.97 | 10.50 ± 14.94 | 1.91±0.75 |
| Alb | g/L | 38.25±5.17 | 38.71±6.36 | 29.47±5.85 | 38.19 ± 6.78 | 45.07±2.36 |
| WBC | 10^9/L | 9.64±2.89 | 19.29±5.35 | 9.94±3.57 | 10.81 ± 6.30 | 5.93±1.59 |
| Platelet | 10^9/L | 197.65±78.36 | 269.89±115.74 | 191.17±107.46 | 224.31 ± 102.02 | 211.79±46.33 |
| Neutrophil | 10^9/L | 7.52±2.81 | 16.7±4.89 | 8.2±3.35 | 8.53 ± 6.21 | 3.49±1.18 |
| Lymphocyte | 10^9/L | 1.41±1.04 | 1.33±0.88 | 1.01±0.4 | 1.37 ± 0.88 | 1.93±0.56 |
| Monocyte | 10^9/L | 0.61±0.25 | 1.17±0.51 | 0.66±0.35 | 0.72 ± 0.40 | 0.33±0.11 |
| RDW-CV | % | 12.93±0.93 | 13.2±1.28 | 16.01±2.17 | 13.88 ± 1.81 | 13.08±0.75 |
| RDW-SD | fL | 42.79±3.33 | 42.52±3.78 | 52.07±8.17 | 45.38 ± 6.80 | 42±2.37 |
| pH | unitless | 7.43±0.06 | 7.42±0.06 | 7.42±0.1 | 7.43 ± 0.08 | - |
| PCO2 | mmHg | 36.92±8.54 | 35.44±8.47 | 37.08±12.69 | 33.80 ± 6.89 | - |
| PO2 | mmHg | 115.04±48.51 | 122.42±62.89 | 125.91±77.58 | 106.25 ± 57.57 | - |
| Lactate | mmol/L | 1.56±1.47 | 2.02±1.32 | 2.06±2.58 | 1.55 ± 1.08 | - |
| SaO2 | % | 97.69±6.63 | 98.12±3.06 | 97.23±4.98 | 97.78 ± 3.58 | - |

**Notes.** Values are mean ± SD or median (IQR), as indicated. IL-6, PCT, CRP were summarized using median (IQR). The other variables were summarized using mean ± SD.

**Table S3** Laboratory distributions in the external validation cohort

| Variable | Unit | Injury level | | | Non-trauma (n=28) | Health  (n=61) |
| --- | --- | --- | --- | --- | --- | --- |
|  |  | Mild (n=211) | Moderate (n=18) | Severe (n=31) |  |  |
| IL-6 | pg/mL | 19.75  (9.26-46.25) | 47.00  (17.51-80.47) | 75.67  (21.69-469.70) | 35.48  (12.91-90.25) | 4.39  (3.55-5.15) |
| PCT | ng/mL | 0.06  (0.04-0.13) | 0.08  (0.04-0.31) | 0.18  (0.09-0.63) | 0.16  (0.06-0.45) | 0.03  (0.03-0.05) |
| CRP | mg/L | 10.60  (3.21-24.09) | 9.93  (1.36-40.83) | 61.18  (34.47-103.46) | 29.50  (3.72-121.00) | 0.84  (0.39-2.25) |
| LMR | ratio | 3.03 ± 2.13 | 1.08 ± 0.88 | 2.06 ± 1.18 | 2.24 ± 1.21 | 5.81 ± 1.83 |
| PLR | ratio | 173.75 ± 110.60 | 656.50 ± 558.68 | 302.46 ± 154.11 | 175.15 ± 125.65 | 108.14 ± 32.58 |
| NLR | ratio | 6.81 ± 6.67 | 31.58 ± 68.77 | 12.98 ± 12.63 | 7.81 ± 8.23 | 1.90 ± 0.95 |
| Alb | g/L | 38.98 ± 4.83 | 40.33 ± 4.42 | 34.02 ± 5.06 | 36.73 ± 5.52 | 44.01 ± 2.70 |
| WBC | 10^9/L | 8.70 ± 2.84 | 16.95 ± 7.46 | 9.14 ± 3.54 | 9.76 ± 5.09 | 6.19 ± 1.84 |
| Platelet | 10^9/L | 195.46 ± 70.12 | 269.28 ± 96.94 | 205.48 ± 91.07 | 189.52 ± 67.87 | 206.15 ± 49.92 |
| Neutrophil | 10^9/L | 6.69 ± 2.81 | 14.77 ± 7.04 | 7.80 ± 3.51 | 7.39 ± 4.73 | 3.59 ± 1.38 |
| Lymphocyte | 10^9/L | 1.42 ± 0.87 | 1.26 ± 0.74 | 0.86 ± 0.55 | 1.45 ± 0.85 | 2.03 ± 0.66 |
| Monocyte | 10^9/L | 0.51 ± 0.20 | 1.89 ± 1.98 | 0.46 ± 0.19 | 0.75 ± 0.51 | 0.37 ± 0.13 |
| RDW-CV | % | 13.27 ± 1.12 | 13.76 ± 1.20 | 16.60 ± 3.36 | 14.20 ± 2.30 | 12.11 ± 0.76 |
| RDW-SD | fL | 45.07 ± 3.81 | 45.38 ± 2.38 | 55.25 ± 7.72 | 46.30 ± 8.75 | 41.32 ± 2.47 |

**Notes.** Values are mean ± SD or median (IQR), as indicated. IL-6, PCT, CRP were summarized using median (IQR). The other variables were summarized using mean ± SD.

**Figure S2** Hematological and routine blood biomarker profiles across development-cohort groups (a) Albumin; (b) WBC; (c) Platelet; (d) Neutrophil; (e) Lymphocyte; (f) Monocytes; (g) PLR; (h) NLR; (i) LMR; (j) RDW-CV; (k) RDW-SD. Overall differences among groups were assessed using the Kruskal–Wallis test, with the corresponding P value shown in each panel.

**Figure S3** Blood-gas biomarker profiles across development-cohort groups (a) pH; (b) pCO2; (c) pO2; (d) Lactate; (e) SaO2. The healthy reference group had no available blood-gas measurements; its position is retained on the x axis, but no observations or boxplots are displayed. Overall differences among groups with available data were assessed using the Kruskal–Wallis test, with the corresponding P value shown in each panel.

**Figure S4** Inflammatory biomarker profiles across development-cohort groups (a) CRP; (b) PCT; (c) IL-6. Overall differences among groups were assessed using the Kruskal–Wallis test, with the corresponding P value shown in each panel.

**Figure S5** The screening path of the LASSO regression model in Context B.

**Table S4** Model specifications used for internal comparison

| **Model** | **R implementation** | **Specification used in the 10-model internal-holdout comparison** | **Regularization or early stopping** |
| --- | --- | --- | --- |
| LightGBM | lightgbm::lgb.train | objective=multiclass; num_class=3; learning_rate=0.05; num_leaves=15; max_depth=-1; min_data_in_leaf=15; feature_fraction=0.80; bagging_fraction=0.80; bagging_freq=1; lambda_l2=1; nrounds=200; deterministic=TRUE; seed=2026 | L2=1; no early stopping |
| XGBoost | xgboost::xgboost | objective=multi:softprob; nrounds=200; max_depth=3; learning_rate=0.05; subsample=0.80; colsample_bytree=0.80; seed=2026; nthreads=1 | No explicit penalty beyond package defaults; no early stopping |
| SVM | e1071::svm | radial kernel; probability=TRUE; scale=FALSE because fold/training-derived standardization was performed separately; cost and gamma used package defaults in the fixed comparison | Margin regularization intrinsic to SVM; no early stopping |
| RF | randomForest::randomForest | ntree=500; mtry=floor(sqrt(10))=3; importance=TRUE; other arguments used package defaults | Not applicable; no early stopping |
| KNN | custom Euclidean KNN | k=max(3,floor(sqrt(399)))=19, forced odd; probabilities were neighbour class proportions | Not applicable |
| Naive Bayes | naivebayes::naive_bayes | usekernel=TRUE; remaining arguments used package defaults | Not applicable |
| Decision tree | rpart::rpart | method=class; cp=0.01; remaining control arguments used package defaults | Cost-complexity pruning cp=0.01; no early stopping |
| Bagged tree | ipred::bagging | nbagg=100; coob=TRUE; base-tree minsplit=10 and cp=0 | No shrinkage; no early stopping |
| Multinomial LR | nnet::multinom | 10-feature final model; trace=FALSE; MaxNWts=10000; maxit=1000; no penalty (decay not specified) | Unregularized final fit; no early stopping |
| Ordinal LR | MASS::polr | method=logistic; Hess=TRUE; ordered outcome Mild<Moderate<Severe; remaining arguments used package defaults | No penalty; no early stopping |

**Table S5** Class-specific performance of the final multinomial logistic regression model in the internal holdout and external validation cohorts

| Cohort | Class | Sensitivity | Specificity | Precision | F1-score | One-vs-rest AUC |
| --- | --- | --- | --- | --- | --- | --- |
| Internal | Mild | 0.934  (0.841-0.982) | 0.950  (0.831-0.994) | 0.966  (0.883-0.996) | 0.950  (0.906-0.985) | 0.984  (0.963-0.998) |
| Internal | Moderate | 0.920  (0.740-0.990) | 0.961  (0.889-0.992) | 0.885  (0.698-0.976) | 0.902  (0.800-0.979) | 0.987  (0.966-0.999) |
| Internal | Severe | 1.000  (0.782-1.000) | 0.988  (0.937-1.000) | 0.938  (0.698-0.998) | 0.968  (0.875-1.000) | 0.999  (0.995-1.000) |
| External | Mild | 0.981  (0.952-0.995) | 0.939  (0.831-0.987) | 0.986  (0.959-0.997) | 0.983  (0.969-0.995) | 0.989  (0.976-0.998) |
| External | Moderate | 0.944  (0.727-0.999) | 0.988  (0.964-0.997) | 0.850  (0.621-0.968) | 0.895  (0.769-0.978) | 0.998  (0.994-1.000) |
| External | Severe | 0.935  (0.786-0.992) | 0.996  (0.976-1.000) | 0.967  (0.828-0.999) | 0.951  (0.885-1.000) | 0.993  (0.984-1.000) |

*Values are presented as estimates with 95% confidence intervals. class-Specific sensitivity, specificity, and precision were accompanied by two-sided Clopper–Pearson exact binomial confidence intervals calculated from the corresponding confusion-matrix counts. Confidence intervals for class-specific F1, and one-versus-rest AUC were estimated using 2,000 patient-level nonparametric bootstrap resamples. The internal holdout cohort was used during staged model selection and should not be interpreted as an independent evaluation cohort of the finally selected model.

**Figure S6** Confusion matrices comparing the 10-feature and 16-feature multinomial logistic regression models in the fixed internal holdout set.

**Figure S7** Calibration of the final 10-feature multinomial logistic regression model. The wide and asymmetric confidence intervals for some class-specific calibration intercepts and slopes, particularly for the external moderate class, reflect the small number of events and instability of one-versus-rest logistic calibration under sparse bootstrap resamples.

**Table S6** Calibration of the final multinomial model

| **Cohort** | **Class** | **Multiclass Brier score (95% CI)** | **Calibration intercept (95% CI)** | **Calibration slope (95% CI)** |
| --- | --- | --- | --- | --- |
| Internal | Mild | 0.106 (0.037-0.194) | 0.590 (-0.498-1.690) | 0.285 (0.201-0.945) |
| Internal | Moderate | 0.106 (0.037-0.194) | -0.471 (-1.438-1.792) | 0.275 (0.188-1.859) |
| Internal | Severe | 0.106 (0.037-0.194) | -7.681 (-87.232-4.811) | 1.493 (0.722-13.078) |
| External | Mild | 0.055 (0.023-0.092) | 0.525 (-0.169-1.183) | 0.309 (0.239-0.589) |
| External | Moderate | 0.055 (0.023-0.092) | -1.357 (-159.206-0.328) | 0.327 (0.251-98.804) |
| External | Severe | 0.055 (0.023-0.092) | -0.048 (-0.974-1.793) | 0.340 (0.255-18.358) |

**Notes.** Calibration was assessed using the original unrecalibrated probabilities. Intercepts and slopes were estimated in one-versus-rest logistic calibration models. Confidence intervals were obtained from 2,000 class-stratified bootstrap resamples.

**Figure S8** SMOTE sensitivity analysis for the 10-feature multinomial logistic regression model (A) Internal-holdout performance across the original training distribution (No SMOTE) and moderate, strong and full SMOTE conditions; (B) Maximum absolute changes in standardized model coefficients and patient-level predicted probabilities relative to the no-SMOTE model; (C) Probability-sensitive performance measures across SMOTE conditions.

**Trauma versus non-trauma discrimination**

Methods: The development clinical-comparator split was retained without modification (training n=471; internal holdout n=120). Trauma was treated as the positive event. The final multinomial logistic regression framework was used with two outcome classes, reducing to ordinary logistic regression. Models were trained with training-only standardization, fixed seed 2026, and a fixed 0.50 classification threshold. A routine eight-variable panel was compared with the addition of CRP, PCT, or IL-6 individually and with all three inflammatory markers. Decision-curve analysis evaluated threshold probabilities from 0.05 to 0.95.

Results: IL-6 produced the largest single-marker improvement in classification performance. The three-marker panel achieved the highest AUC but did not exceed the IL-6-only panel for balanced accuracy, macro-F1, or MCC. The routine and inflammation-enhanced models exceeded both default decision strategies at 54 and 73 of 91 internal thresholds, respectively.

**Table S7** Sensitivity analysis of CRP, PCT, and IL-6 in trauma versus non-trauma classification

| **Panel** | **Balanced accuracy** | **Macro-F1** | **MCC** | **AUC** | **Non-trauma recall** |
| --- | --- | --- | --- | --- | --- |
| Routine 8 | 0.605 | 0.639 | 0.428 | 0.681 | 0.211 |
| + CRP | 0.605 | 0.639 | 0.428 | 0.687 | 0.211 |
| + PCT | 0.574 | 0.591 | 0.301 | 0.715 | 0.158 |
| + IL-6 | 0.632 | 0.676 | 0.481 | 0.684 | 0.263 |
| + CRP + PCT + IL-6 | 0.627 | 0.665 | 0.424 | 0.722 | 0.263 |

Note: Routine 8 comprised age, albumin, platelet count, neutrophil count, monocyte count, PLR, RDW-CV, and RDW-SD.

**Figure S9** Decision-curve using the final logistic regression framework for trauma versus non-trauma classification with trauma as the event. Net benefit is shown for the routine eight-variable model, the inflammation-enhanced model adding CRP, PCT, and IL-6, and treat-all and treat-none strategies across threshold probabilities of 0.05–0.95.

**Table S8** Incremental predictive value of CRP and PCT in the multinomial trauma severity model

| **Panel** | **Cohort** | **Balanced accuracy** | **Macro-F1** | **MCC** | **Macro-AUC** | **Multiclass Brier score** | **Severe sensitivity** |
| --- | --- | --- | --- | --- | --- | --- | --- |
| Routine 8 | Internal | 0.883 | 0.876 | 0.806 | 0.985 | 0.136 | 0.867 |
| + CRP | Internal | 0.924 | 0.917 | 0.859 | 0.989 | 0.103 | 0.933 |
| + PCT | Internal | 0.946 | 0.931 | 0.878 | 0.989 | 0.111 | 1.000 |
| + CRP +PCT | Internal | 0.951 | 0.940 | 0.894 | 0.990 | 0.106 | 1.000 |
| Routine 8 | External | 0.841 | 0.837 | 0.746 | 0.980 | 0.113 | 0.677 |
| + CRP | External | 0.929 | 0.918 | 0.870 | 0.992 | 0.065 | 0.871 |
| + PCT | External | 0.860 | 0.847 | 0.761 | 0.983 | 0.119 | 0.677 |
| + CRP +PCT | External | 0.954 | 0.943 | 0.917 | 0.993 | 0.055 | 0.935 |
